# Sublethal Caspase-8 Activation Drives Radiation-Induced Genetic Instability and Oncogenic Transformation Through Endonuclease G and Non-Canonical NF-κB Signaling

**DOI:** 10.3390/ijms27167398

**Published:** 2026-08-19

**Authors:** Chenchen Zhu, Xiaoxiao Li, Chuan-Yuan Li, Renwei Liu, Yifei Wang

**Affiliations:** 1 College of Life Science and Technology, Jinan University, Guangzhou 510632, China; renwei.liu@bebettermed.com (R.L.); twang-yf@163.com (Y.W.); 2 Department of Dermatology, Duke University School of Medicine, Durham, NC 27710, USA; lixiaoxiao327@163.com (X.L.); chuan.li@duke.edu (C.-Y.L.)

**Keywords:** caspase-8, radiation, genomic instability, endonuclease G, non-canonical NF-κB, oncogenic transformation

## Abstract

Apoptosis is traditionally considered a definitive barrier against oncogenesis, as caspase activation typically eliminates damaged and genetically unstable cells. Here, we report that caspase-8, an initiator of extrinsic apoptosis, paradoxically promotes genetic instability and carcinogenesis following exposure to radiation. We observed that a substantial fraction of mammalian cells exposed to ionizing radiation can survive despite caspase-8 activation. This sublethal activation of caspase-8 facilitated persistent DNA damage, which was attenuated by the expression of a dominant-negative caspase-8 (C360A, Casp8DN) or short hairpin RNA (shRNA)-mediated knockdown of caspase-8 in mammalian cells. The facilitative role of caspase-8 in radiation-induced genomic instability was further validated in caspase-8 heterozygous mice. Moreover, inhibition of caspase-8 abolished iron-ion radiation-induced oncogenic transformation in both soft agar and nude mice. Mechanistically, sublethal caspase-8 activation promoted the nuclear translocation of mitochondrial endonuclease G and persistent DNA damage, accompanied by activation of non-canonical nuclear factor κB (NF-κB) signaling. Collectively, our findings demonstrate that caspase-8 can act as a causative driver of radiation-induced malignancy, challenging the dogma of caspases as universal anticancer barriers and providing important insights into the long-term health risks associated with space radiation and radiotherapy.

## 1. Introduction

Apoptosis, or programmed cell death, is a fundamental form of regulated cell death in multicellular organisms. By eliminating damaged or potentially deleterious cells, it maintains tissue homeostasis and constitutes a critical barrier against tumorigenesis [1]. The caspase proteolytic cascade is the central driver of apoptosis. Caspase-8, an initiator caspase, initiates extrinsic apoptosis via death receptor family members, including tumor necrosis factor (TNF)-related apoptosis-inducing ligand (TRAIL) receptors and CD95/FAS [2,3,4]. Upon ligand binding, death receptors recruit and activate caspase-8 through dimerization facilitated by adaptor proteins like FAS-associated death domain protein (FADD) [5,6]. Active caspase-8 then cleaves substrates at its consensus recognition motif, isoleucine-glutamic acid-threonine-aspartic acid (IETD) [7] and cascades to activate the caspase-3 and caspase-7 effectors [8], leading to widespread cleavage of caspase substrates and rapid apoptotic cell death.

However, the emergence of phenotypes that cannot be attributed solely to defective apoptosis has challenged this classical paradigm [9,10,11]. In mice, caspase-8 deficiency during development causes embryonic lethality due to profound vascular and neural tube defects independent of its pro-apoptotic activity [9,12,13], which can be rescued by deletion of the *RIPK3* or *MLKL* gene [14,15]. Similarly, mice expressing an enzymatically inactive caspase-8 (*Casp8*^C362S/C362S^) die embryonically due to aberrant necroptosis and pyroptosis [16]; *MLKL* deficiency rescues embryonic lethality but unexpectedly results in perinatal death, fully corrected in *Casp8*^C362S/C362S^*MLKL*^−/−^*Asc*^−/−^ or *Casp8*^C362S/C362S^*MLKL*^−/−^*Casp1*^−/−^ mice [17]. Beyond its role in cell death regulation, caspase-8 functions as a pleiotropic signaling node essential for organism viability, supporting T-cell receptor-driven proliferation and maintaining intestinal epithelial barrier integrity via suppression of the RIPK3-MLKL axis [10,18,19]. Collectively, these findings reframe caspase-8 not merely as a death trigger but as a critical molecular switch for tissue homeostasis [17].

This functional duality necessitates a reevaluation of caspase-8’s role in oncogenesis. While loss-of-function mutations or epigenetic silencing in cancers such as neuroblastomas and medulloblastomas support its tumor-suppressive role [20,21], caspase-8 can paradoxically promote malignancy. In small-cell lung carcinoma, caspase-8 deficiency enhances metastasis and therapy resistance via necroptosis-driven inflammation [20]. Conversely, apoptotic machinery can be hijacked to support tumor progression; for example, CD95/FAS signaling drives glioblastoma invasion through PI3K/AKT and nuclear factor κB (NF-κB) activation [22,23]. Furthermore, deletion of *caspase-3/7* impairs physiological processes like liver regeneration [24] while reducing tumor stemness [25], whereas elevated activated caspase-3 correlates with recurrence and poor prognosis [26]. A transformative concept in this field is “minority mitochondrial outer membrane permeabilization (MOMP)”, in which mitochondrial outer membrane permeabilization occurs in only a subset of mitochondria, resulting in limited, sublethal caspase activation. Studies have shown that limited mitochondrial outer membrane permeabilization, induced by sublethal doses of apoptotic stimuli, promotes DNA damage and genetic instability—outcomes that are contrary to conventional wisdom [27,28]. However, whether initiator caspases of the extrinsic pathway, particularly caspase-8, can similarly undergo sublethal activation to cross-talk with mitochondrial elements and drive genomic instability remains largely unexplored.

Different types of ionizing radiation, including low-linear energy transfer (LET) X-rays and high-LET heavy ions, can induce DNA damage and persistent genomic instability in surviving cells. High-LET ^56^Fe ions are of particular interest because they can induce more complex DNA damage and exhibit greater biological effectiveness [29,30] while also representing an important component of the deep-space radiation environment. Although these genomically unstable cells can survive long-term, the underlying mechanisms remain elusive. In this study, we examined our hypothesis that caspase-8 facilitates oncogenic transformation by inducing genetic instability in irradiated cells that survived. We demonstrated that sublethal caspase-8 activation promotes persistent genomic instability and endonuclease G (EndoG) nuclear translocation, accompanied by activation of non-canonical NF-κB signaling, which may provide a survival advantage to genomically damaged cells and thereby facilitate malignant transformation. Our findings identify caspase-8 as a causative driver of radiation-induced malignancy, offering vital insights into the long-term health risks associated with space radiation and radiotherapy.

## 2. Results

### 2.1. Ionizing Radiation Induces Sublethal Activation of Caspase-8

**Figure 1:**
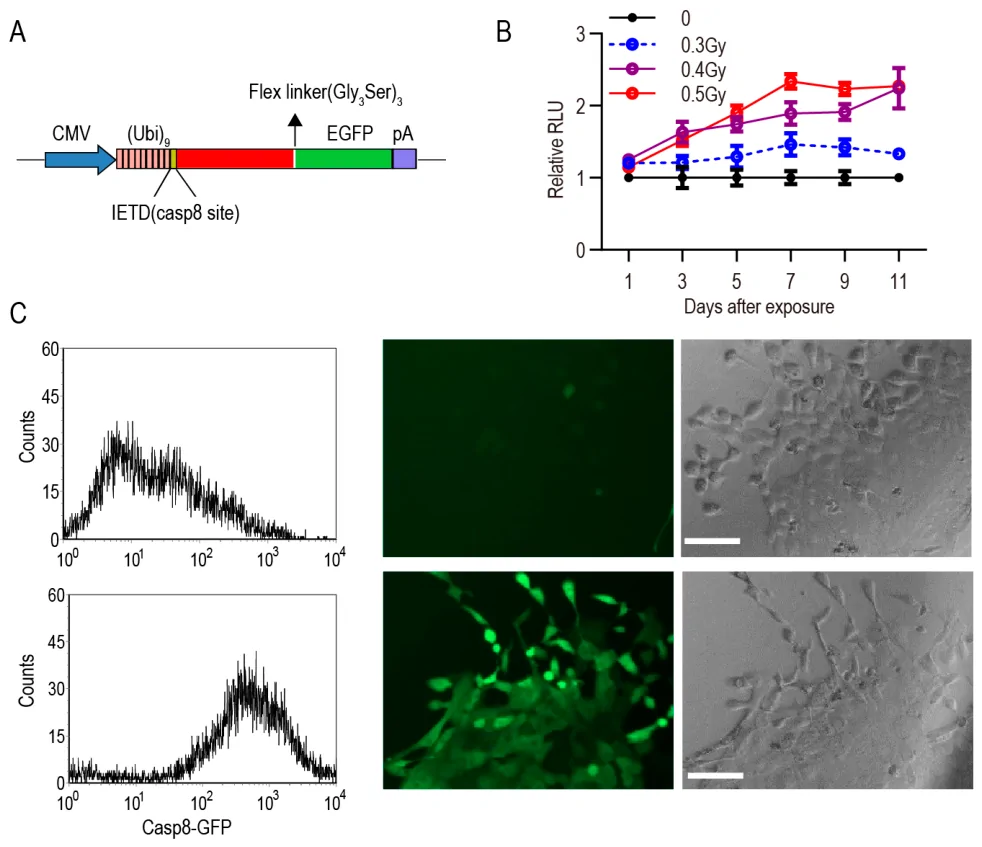
Sublethal activation of caspase-8 in MCF10A cells exposed to radiation. (**A**) Diagram of the caspase-8 reporter gene (Casp8-GL). Nine-ubiquitin domain, (Ubi)9, serves as a proteasome recognition signal to induce rapid degradation of the luciferase-EGFP fusion protein. The caspase-8 cleavage site (IETD) was inserted between the polyubiquitin domain and the fusion protein. Stress-induced caspase-8 activation cleaves at the IETD site, which stabilizes the luciferase-EGFP fusion protein for detection. (**B**) Caspase-8 reporter luciferase activity in MCF10A cells irradiated with various doses of iron ions. RLU stands for relative luminescence unit. *n* = 3 biological replicates; error bars represent standard error of the mean (SEM). (**C**) Cell populations with low (**top panel**) and high (**lower panel**) EGFP expression from iron-ion-irradiated (0.5 Gy) MCF10A cells were separated by flow cytometry at 10 days after irradiation. Scale bars represent 50 μm.

Radiation-induced activation of apoptosis in cells is traditionally considered an irreversible commitment to eliminate damaged host cells, under the assumption that any cells undergoing caspase cascade activation are destined to die. However, experimental data on the relationship between caspase-8 (Casp8) activation and the fate of irradiated cells are lacking. To monitor the kinetics and dynamics of caspase-8 activation in irradiated mammalian cells, we developed a novel non-invasive caspase-8 reporter (Figure 1A). In this reporter, the luciferase–GFP fusion gene is linked to a polyubiquitin domain, rendering the fusion protein highly unstable and rapidly degraded by the proteasome. A caspase-8 cleavage site (IETD) [31] was inserted between the GFP and polyubiquitin domains (Casp8-GL). Upon activation of caspase-8, the polyubiquitin domain is cleaved, resulting in stabilization of the luciferase–GFP protein. Using this system, we systematically investigated the fate of cells with Casp8 activation. Human mammary epithelial cells (MCF10A) were transduced with the Casp8-GL reporter and then irradiated with low-dose high-energy iron ions (^56^Fe, 0.3–0.5 Gy). Our results showed that radiation induced significant activation of the caspase-8 reporter in a dose-dependent and persistent manner (Figure 1B). To validate these findings across different radiation qualities, we also exposed cells to X-ray irradiation. Consistent with our reporter data, a corresponding increase in cleaved caspase-8 was observed at the protein level (Figure S1A). Irradiated cells were sorted by flow cytometry based on their cellular GFP levels (Figure 1C). Notably, those cells with high GFP expression maintained typical adherent morphology under microscopic observation (Figure 1C, lower panels; Figure S1B). Importantly, this subpopulation of cells remained viable despite the activation of caspase-8. This observation is consistent with previous studies showing that a fraction of MCF10A cells can survive substantial radiation exposure and retain clonogenic capacity, including radioresistant subpopulations that persist following high-dose irradiation [32,33]. Collectively, these data demonstrate that MCF10A cells can tolerate specific levels of caspase-8 activation without undergoing programmed cell death, suggesting a sublethal, non-canonical mode of radiation-induced caspase-8 activation.

### 2.2. Caspase-8 Plays a Facilitative Role in Radiation-Induced Persistent DNA Damage in Surviving Cells

**Figure 2:**
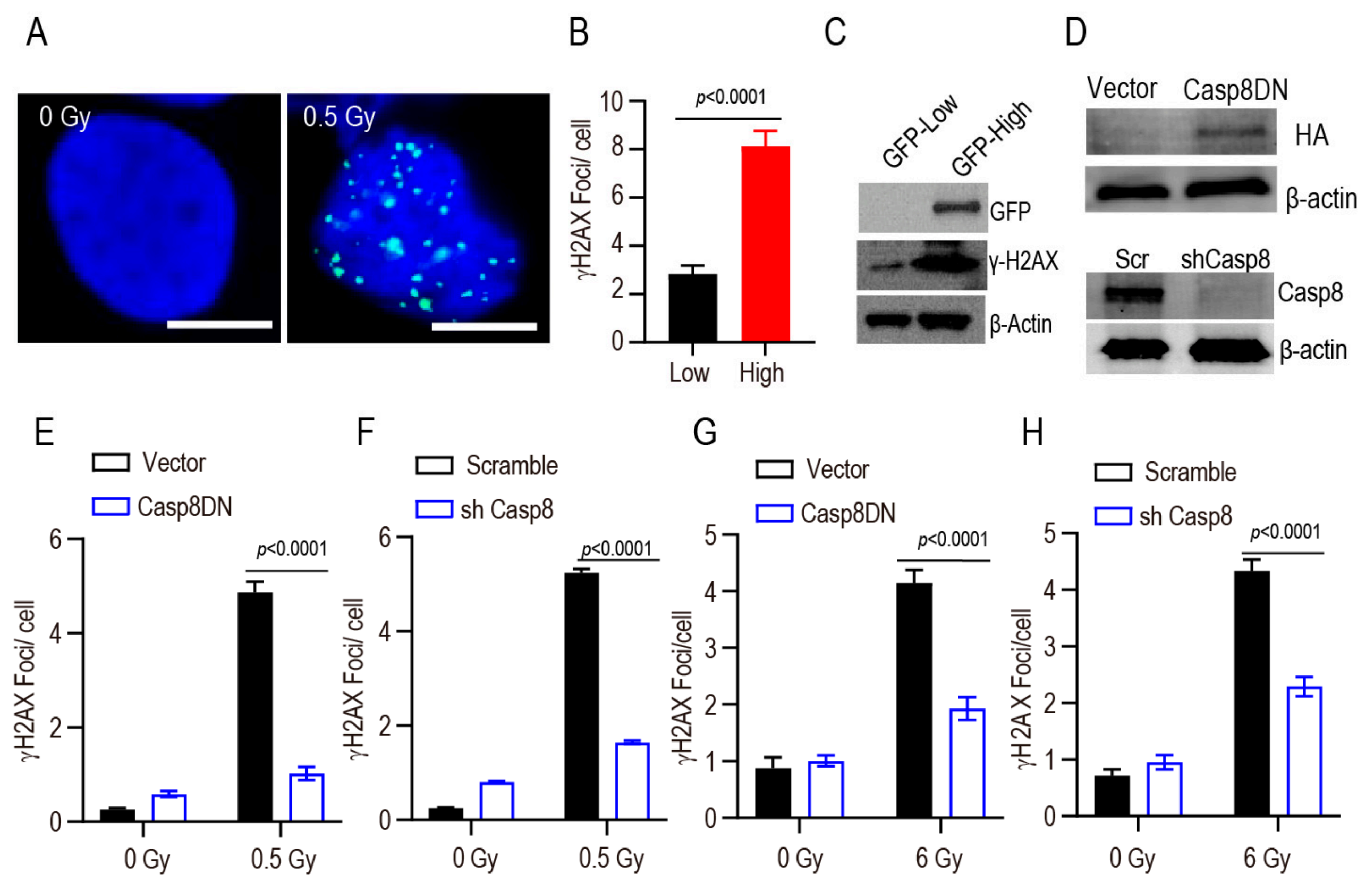
Caspase-8 activation induces sustained γH2AX foci formation in irradiated cells. (**A**) Immunofluorescence (IF) staining showed representative γH2AX foci in non-irradiated (0 Gy) and irradiated (0.5 Gy) MCF10A cells. Blue indicates nuclei, and green indicates γH2AX foci. Scale bars represent 10 μm. (**B**,**C**) Average number of γH2AX foci determined by IF (**B**) and Western blot analysis of γH2AX expression (**C**) in EGFP-low and EGFP-high-expressing MCF10A-Casp8-GL cells 14 days after 0.5 Gy iron-ion radiation exposure. (**D**) Western blot showing ectopic expression of HA-tagged dominant-negative caspase-8 (Casp8DN) in MCF10A cells (**upper**), as well as shRNA-mediated knockdown of caspase-8 expression in MCF10A cells transduced with caspase-8 shRNA (shCasp8) (**lower**). (**E**) Dominant-negative caspase-8 attenuated γH2AX foci formation in 0.5 Gy iron-ion-irradiated MCF10A cells. The data show the average number of γH2AX foci at 20 days after irradiation. (**F**) The effect of caspase-8 knockdown on γH2AX foci formation in 0.5 Gy iron-ion-irradiated MCF10A cells. The data show the average number of γH2AX foci at 8 days after irradiation. (**G**,**H**) Attenuation of caspase-8 function, achieved either by expression of its dominant-negative form (Casp8DN) (**G**) or by shRNA-mediated knockdown (shCasp8) (**H**), significantly reduced the average number of γH2AX foci in 6 Gy X-ray-irradiated MCF10A cells at 10 days post-irradiation. Data are presented as mean ± SEM (*n* = 3 biological replicates per group (**B**,**E**–**H**)). *p* values were determined using Student’s *t*-test in (**B**,**E**–**H**).

Irradiated cells are typically assumed to either recover via endogenous DNA damage repair systems or eliminate themselves through the activation of apoptotic cascades that fragment DNA shortly after exposure. Caspase-8 is a well-known initiator of apoptotic cell death. Therefore, our observation of its sublethal activation in irradiated cells raises an intriguing question: what is the long-term impact of this event on the genomic stability of surviving cells? We carried out experiments to examine DNA damage in cells that survived despite caspase-8 activation. Our data showed that irradiated cells exhibited significantly higher levels of γH2AX foci-a hallmark of DNA double-strand breaks (DSBs)-compared with non-irradiated cells (Figure 2A) at two weeks post-irradiation. Furthermore, cells with higher levels of Casp8-GFP reporter activity had significantly higher numbers of γH2AX foci compared with those with lower levels of reporter activity (Figure 2B,C).

To provide definitive proof that the cleavage activity of caspase-8 is necessary for persistent DNA damage, we constructed a lentiviral vector encoding a dominant-negative form of caspase-8 (Casp8DN). The mutant carries a single amino acid change (C360A) that disables its proteolytic function [34]. MCF10A cells transduced with Casp8DN (Figure 2D) were then irradiated. Our data showed that the number of γH2AX foci was significantly lower in irradiated MCF10A cells expressing Casp8DN after iron-ion or X-ray irradiation (Figure 2E,G) compared with vector controls. This causative role was further validated using short hairpin RNA (shRNA)-mediated knockdown of caspase-8 (Figure 2D), which similarly reduced radiation-induced γH2AX foci compared to scrambled controls (Figure 2F,H). Thus, our data demonstrated that caspase-8 plays a key role in facilitating radiation-induced persistent DNA damage.

**Figure 3:**
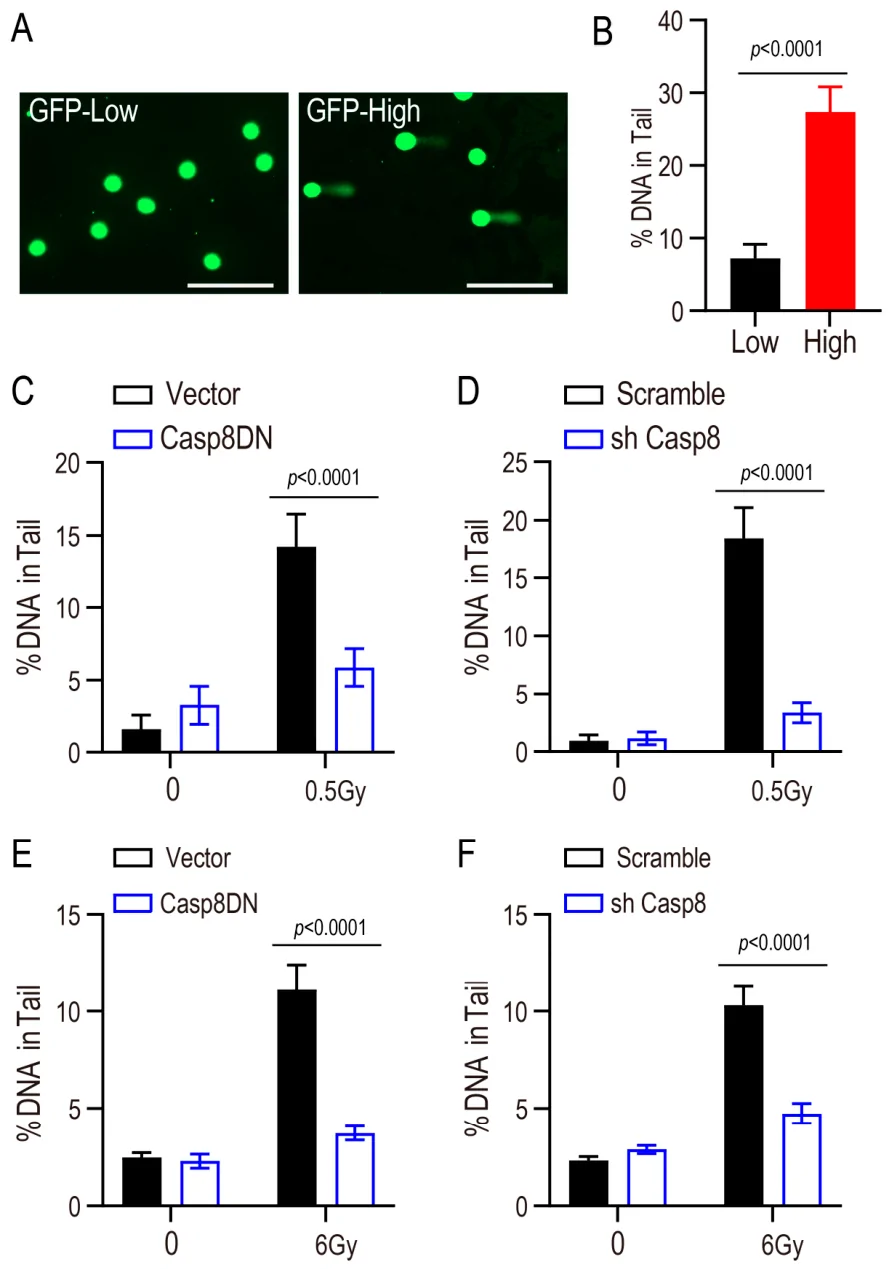
Caspase-8 activation plays a causative role in radiation-induced DNA damage as determined by the comet assay. (**A**) Representative images of irradiated MCF10A cells with low and high caspase-8 activities when run through electrophoresis during the comet assay. Scale bars represent 50 μm. (**B**) The percentage of DNA in the comet tail of MCF10A cells with low and high caspase-8 activities at 20 days after 0.5 Gy iron-ion radiation exposure. (**C**,**D**) Silencing of caspase-8 activity by dominant-negative caspase-8 expression (**C**) or shRNA-mediated knockdown (**D**) reduced the percentage of DNA in the comet tail in 0.5 Gy iron-ion-irradiated MCF10A cells. The data show the percentage of DNA in the comet tail at 20 days (**C**) and 8 days (**D**) after irradiation. (**E**,**F**) Silencing of caspase-8 activity by dominant-negative caspase-8 (**E**) and shRNA (**F**) reduced the percentage of DNA in the comet tail of MCF10A cells exposed to 6 Gy X-ray irradiation. The data show the percentage of DNA in the comet tail at 15 days post-irradiation. Data are presented as mean ± SEM (*n* = 3 biological replicates per group (**B**–**F**)). *p* values were determined using Student’s *t*-test in (**B**–**F**).

We further determined the role of caspase-8 in radiation-induced persistent DNA damage by performing the comet assay, another approach to measure DNA strand breaks (Figure 3A). The sizes of the “comet tails” represent the extent of DNA damage. As shown in Figure 3B, surviving cells with higher levels of Casp8-GL reporter activity exhibited a significantly higher percentage of DNA in the comet tail, which means higher levels of DNA damage, when compared with those with lower levels of reporter activity. Crucially, inhibiting caspase-8 activity via Casp8DN (Figure 3C,E) or shRNA (Figure 3D,F) in irradiated MCF10A cells significantly reduced the percentage of DNA in the comet tail compared with that in control cells. Therefore, these findings demonstrate that radiation-induced caspase-8 activation is required to induce persistent, secondary DNA damage in surviving cells long after the initial repair of radiation-induced direct DNA damage.

### 2.3. Caspase-8 Activation Drives Radiation-Induced Chromosomal Aberrations In Vivo

**Figure 4:**
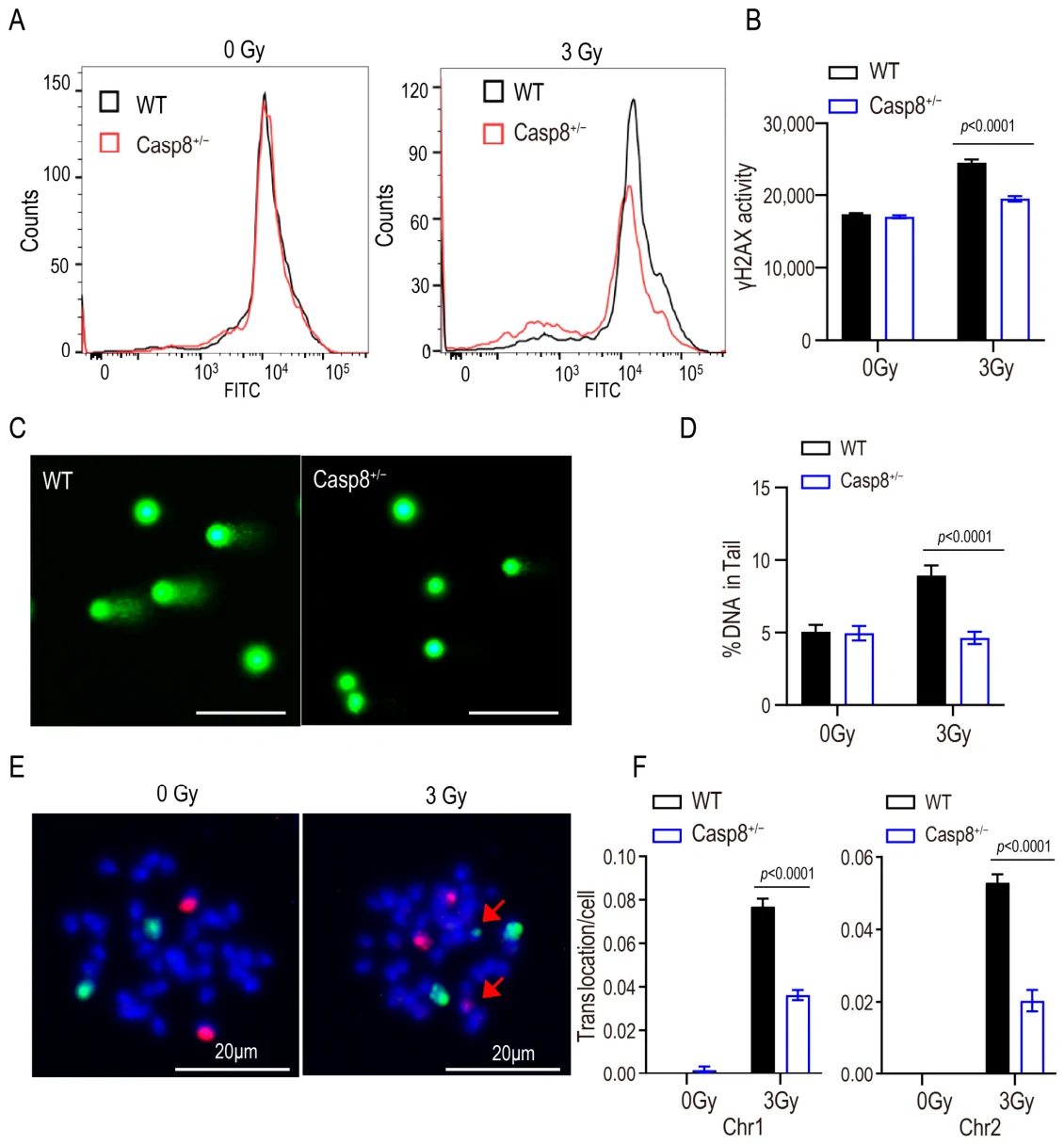
Caspase-8 activation drives radiation-induced chromosomal aberrations in wild-type mice compared to heterozygous *Casp8*^+/−^ mice. (**A**) Flow cytometric analysis of γH2AX expression in bone marrow cells from non-irradiated (**left**) and irradiated (**right**) WT and *Casp8*^+/−^ mice at 6 days after 3 Gy irradiation. (**B**) Quantification of γH2AX fluorescence intensity in bone marrow cells from WT and *Casp8*^+/−^ mice at 6 days after 3 Gy irradiation. (**C**) Representative comet assay images of bone marrow cells from WT and *Casp8*^+/−^ mice at 6 days after 3 Gy X-ray irradiation. Scale bars represent 50 μm. (**D**) Quantification of comet tail DNA percentage in bone marrow cells from WT and *Casp8*^+/−^ mice at 6 days after 3 Gy X-ray irradiation. (**E**) Representative images of whole-chromosome painting for chromosomes 1 (green) and 2 (red). The (**left**) panel shows normal chromosomes; the (**right**) panel shows a cellular chromosome spread with a translocation involving parts of chromosomes 1 and 2 (red arrows) in irradiated mice. (**F**) Quantification of radiation-induced translocations involving chromosomes 1 and 2 in bone marrow cells from WT and *Casp8*^+/−^ mice at 6 days after 3 Gy X-ray irradiation. Data are presented as mean ± SEM (*n* = 3 biological replicates per group (**B**,**D**,**F**)). *p* values were determined using Student’s *t*-test in (**B**,**D**,**F**).

To determine whether caspase-8 mediates persistent DNA damage in vivo, we evaluated radiation-induced genomic instability in caspase-8 heterozygous (*Casp8*^+/−^) mice, as homozygous deletion is embryonically lethal [35]. Following whole-body 3 Gy X-ray irradiation, radiosensitive bone marrow cells were analyzed to assess the in vivo role of caspase-8. Flow cytometry for phosphorylated H2AX (γH2AX) levels revealed that irradiated *Casp8*^+/−^ bone marrow cells displayed significantly lower γH2AX intensity compared to wild-type (WT) controls (Figure 4A,B). Consistent with these γH2AX data, the percentage of DNA in the comet tail was markedly reduced in bone marrow cells from irradiated *Casp8*^+/−^ mice (Figure 4C,D). Furthermore, radiation-induced chromosomal translocations involving chromosomes 1 and 2 were assessed using whole-chromosome painting (Figure 4E). Importantly, bone marrow cells from *Casp8*^+/−^ mice showed significantly fewer radiation-induced translocations involving chromosomes 1 and 2 compared with those from WT mice (Figure 4F). These data demonstrate that caspase-8 facilitates radiation-induced genomic instability in vivo, consistent with our in vitro observations.

### 2.4. Caspase-8 Promotes Oncogenic Transformation of Irradiated MCF10A Cells

**Figure 5:**
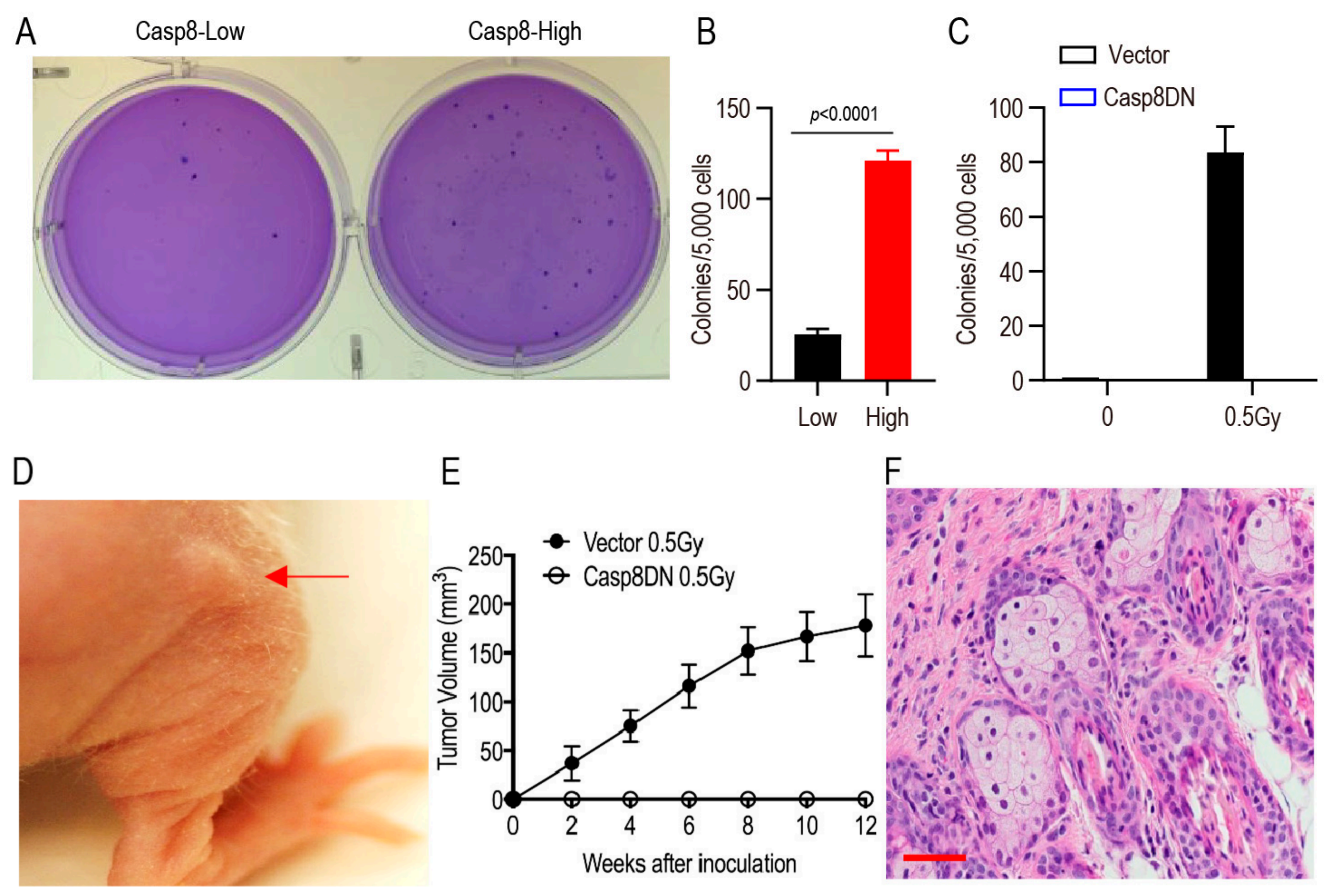
Oncogenic transformation caused by iron-ion-induced caspase-8 activation. (**A**) Representative images of soft agar colony formation in irradiated (0.5 Gy) MCF10A cells with low and high caspase-8 activities. (**B**) Quantification of soft-agar colony formation in MCF10A cells with high and low caspase-8 activities. The iron-ion-irradiated MCF10A cells were sorted for high and low caspase-8 reporter expression. They were then cultured for 2 weeks and seeded in soft agar plates. Data are presented as mean ± SEM; *n* = 3 biological replicates per group. *p* values were determined using Student’s *t*-test. (**C**) The effect of dominant-negative caspase-8 (Casp8DN) expression on the soft agar colony formation ability of iron-ion-irradiated MCF10A cells. Dominant-negative caspase-8 (Casp8DN) expression abolished the soft agar colony formation ability of irradiated MCF10A cells. Data are presented as mean ± SEM, *n* = 3 biological replicates per group. (**D**) A xenograft tumor in nude mice after 3 weeks of inoculation with iron-ion-irradiated (0.5 Gy) MCF10A cells (indicated by the red arrow). (**E**) Tumorigenic ability of iron-ion-irradiated (0.5 Gy) MCF10A cells with vector and dominant-negative caspase-8 (Casp8DN) expression (*n* = 10 per group); 14 days after irradiation, 2 × 10^6^ cells were injected subcutaneously into the hind limb of 6–8-week-old female nude mice. The irradiated parental MCF10A cells can form tumors in nude mice, while Casp8DN expression abolished the tumorigenic ability of irradiated MCF10A cells. (**F**) H&E staining of tissues derived from a xenograft tumor formed from irradiated MCF10A cells in nude mice. Scale bar = 50 μm.

We next asked whether surviving cells that tolerate caspase-8 activation and persistent DNA damage undergo oncogenic transformation. To investigate this, iron-ion-irradiated cells were cultured for two weeks and subsequently plated in soft agar to estimate anchorage-independent colony formation, a hallmark of cellular transformation. Only transformed cells are capable of growing in soft agar in an anchorage-independent manner. Our results showed that surviving cells with high caspase-8 activity had a significantly higher frequency of colony formation compared with cells with low caspase-8 activity (Figure 5A,B). Expression of dominant-negative caspase-8 completely abolished colony formation in irradiated MCF10A cells (Figure 5C), indicating that caspase-8 plays a causative role in radiation-induced oncogenic transformation. We further confirmed this role in nude mice. High-linear energy transfer iron-ion irradiation (0.5 Gy) enabled parental MCF10A cells to form tumors in nude mice, whereas expression of dominant-negative caspase-8 abolished the tumorigenic ability of irradiated MCF10A cells (Figure 5D–F). However, low-linear energy transfer X-ray radiation did not induce oncogenic transformation in MCF10A cells, as evidenced by the absence of soft agar colony formation and tumor formation in nude mice. These data further demonstrated that caspase-8 activity is essential for radiation-induced tumorigenicity in MCF10A cells.

### 2.5. Caspase-8-Dependent EndoG Nuclear Translocation Is Associated with Persistent DNA Damage and Non-Canonical NF-κB Activation

**Figure 6:**
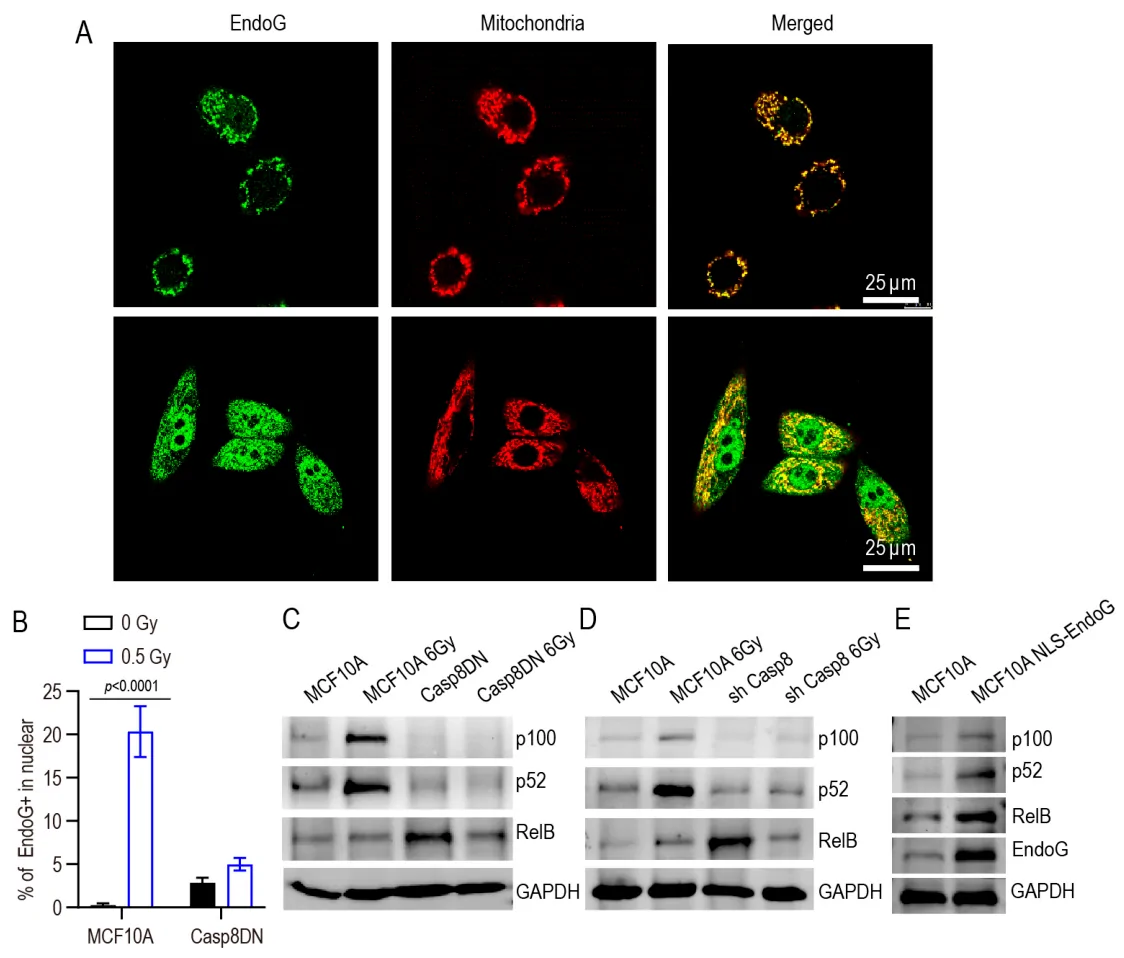
Caspase-8-dependent EndoG nuclear translocation is associated with persistent DNA damage and non-canonical NF-κB activation. (**A**) Immunofluorescence co-staining of EndoG (green) and mitochondria (red) in GFP-low (**top panels**) and GFP-high (**lower panels**) MCF10A cells. For immunofluorescent staining, cells were treated with AutoFluoQuencher for 30 min to quench GFP fluorescence before incubation with primary antibodies. (**B**) Expression of dominant-negative caspase-8 (Casp8DN) attenuates radiation-induced EndoG nuclear translocation. *p* values were determined using Student’s *t*-test. Data are presented as mean ± SEM, *n* = 3 biological replicates per group. (**C**,**D**) Western blot analysis of non-canonical NF-κB pathway components (p100, p52, RelB) in MCF10A cells expressing Casp8DN (**C**) or shCasp8 (**D**) at 10 days post-irradiation (6 Gy). (**E**) Western blot analysis of non-canonical NF-κB pathway components in MCF10A-NLS-EndoG cells.

EndoG is localized in mitochondria under normal conditions and translocates into the nucleus to fragment DNA during apoptosis. Sublethal stresses induce limited mitochondrial outer membrane permeabilization, leading to restricted EndoG translocation and caspase-3 activation without triggering cell death, thereby promoting cellular transformation and tumorigenesis [28,36]. Cells with low caspase-8 reporter activity exhibited predominantly cytoplasmic, perinuclear EndoG localization, co-localizing with mitochondrial markers, with minimal nuclear staining (Figure 6A, top). In contrast, cells with high reporter activity showed a significant increase in nuclear EndoG staining (Figure 6A, bottom), indicating mitochondrial-to-nuclear translocation. This process was dependent on caspase-8 activity, as the expression of dominant-negative caspase-8 (Casp8DN) attenuated EndoG nuclear translocation in iron-ion-irradiated MCF10A cells (Figure 6B). Although the precise mechanistic link remains to be defined, our findings are consistent with the “minority MOMP” paradigm, in which limited mitochondrial outer membrane permeabilization results in sublethal caspase activation without triggering cell death. Given that caspase-8 can cleave BID to promote mitochondrial permeabilization [27,37], BID cleavage may provide a potential mechanistic link between sublethal caspase-8 activation and the restricted release of mitochondrial EndoG observed in our study. This suggests that radiation-induced caspase-8 activity initiates a cascade culminating in the restricted release and nuclear translocation of EndoG. We previously demonstrated that EndoG contributes functionally to persistent radiation-induced DNA damage in MCF10A cells, as EndoG knockdown reduced γH2AX foci and comet tail DNA [28]. To elucidate the downstream pro-survival mechanisms following caspase-8-mediated DNA damage, we performed RNA sequencing (RNA-seq) on sorted GFP-high and GFP-low subpopulations of MCF10A cells. This identified 1372 differentially expressed genes (DEGs), with 566 genes upregulated and 806 downregulated in the GFP-high fraction (Figure S2A). KEGG enrichment analysis further implicated dysregulation of DNA repair mechanisms and activation of the NF-κB signaling pathway (Figure S2B). Specifically, the GFP-high subpopulation exhibited a distinct transcriptomic signature characterized by the significant upregulation of key non-canonical NF-κB components, including *NFKB2* (encoding p100/p52) and *RelB* (Figure S2C), pointing to a preferential activation of this pathway in cells with elevated caspase-8 activity. Consistent with these transcriptomic findings, Western blotting revealed that high caspase-8 activity in irradiated MCF10A cells correlated with the increased expression of the non-canonical NF-κB effectors p100/p52 and RelB (Figure 6C,D), an effect that was abolished by Casp8DN or shRNA-mediated knockdown. Furthermore, expression of an NLS-EndoG fusion in MCF10A cells was sufficient to induce high expression of p52 and RelB (Figure 6E).

**Figure 7:**
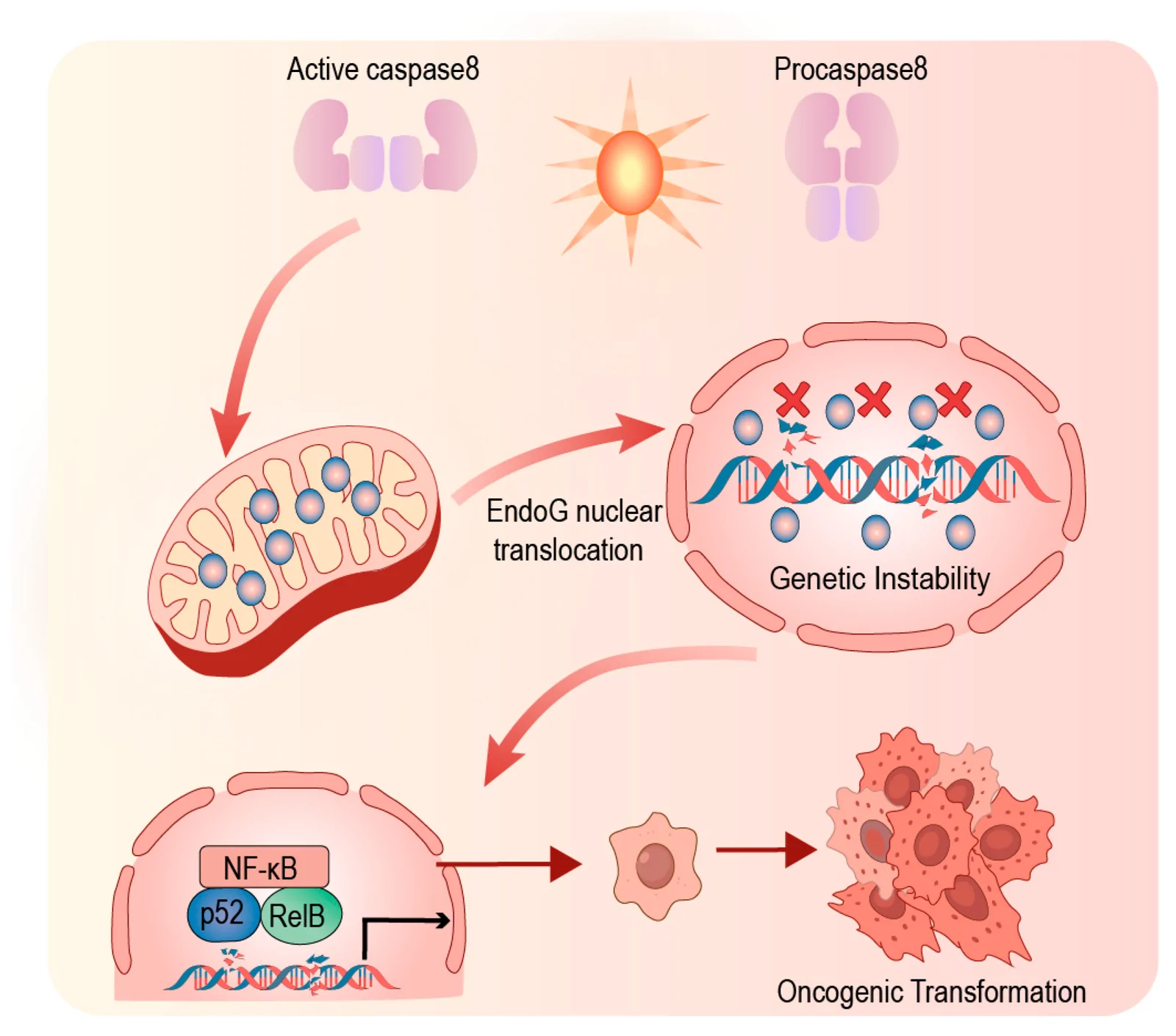
Diagrammatical image illustrates the paradoxical role of caspase-8 in radiation-induced carcinogenesis. Ionizing radiation induces sublethal caspase-8 activation, promoting EndoG translocation from mitochondria to the nucleus and persistent DNA damage. These changes are associated with activation of non-canonical NF-κB signaling, which may provide a survival advantage to genomically damaged cells and thereby facilitate malignant transformation. Arrows indicate promoting effects, whereas crosses indicate DNA damage.

These findings demonstrate that under radiation-induced stress, caspase-8 promotes EndoG nuclear translocation and sustained DNA damage, which are accompanied by activation of the non-canonical NF-κB signaling pathway (p52/RelB). This signaling response may contribute to the persistence and malignant transformation of genomically damaged cells (Figure 7). These findings expand upon the classical paradigm of the “ATM/ATR–NF-κB signaling axis in maintaining tumorigenic potential” [25]. Moreover, they align with previous reports showing that caspase-8 can mediate pro-survival signaling through phosphorylation or by functioning as a scaffold protein [9,38,39].

## 3. Discussion

Caspase activation has traditionally been viewed as a definitive tumor-suppressive mechanism, yet frequent mutations or deletions of apoptotic inducers, such as *CASP8*, in clinical tumor samples suggest otherwise [40,41]. In this study, we demonstrated that ionizing radiation induces sublethal caspase-8 activation, which paradoxically promotes genomic instability and facilitates malignant transformation both in vitro and in vivo. This challenges the conventional apoptosis-centric paradigm, adding a critical new dimension to our understanding of non-apoptotic caspase functions.

Although counterintuitive, our findings are consistent with the essential roles of caspase-8 in maintaining tissue homeostasis and physiological signaling. A core role of caspase-8 is the precise suppression of RIPK1/RIPK3/MLKL-mediated necroptosis, enabling cells to execute high-stress programs such as proliferation and differentiation [14,15,42]. Its enzymatic activity is also indispensable for T-cell receptor-induced NF-κB activation and subsequent clonal expansion [10], as well as precursor cell survival during osteogenesis and myogenesis [9,43,44]. Our current study demonstrates that under radiation-induced genotoxic stress, this pro-survival potential of caspase-8 is hijacked, undergoing a “qualitative transformation” that drives tumorigenesis. Despite caspase-8 activation, a subset of cells survives irradiation and serves as the seed for malignant transformation, aligning with the notion that tumors leverage the non-apoptotic scaffolding activity of caspase-8 to promote survival, migration, and therapeutic resistance [20,45,46,47,48,49].

Under radiation stress, sublethal caspase-8 activation shifts its role from a “guardian” that eliminates damaged cells to a “catalyst” for genomic deterioration. Activated caspase-8 can cleave BID to generate tBID, thereby promoting BAX/BAK-mediated mitochondrial outer membrane permeabilization [50,51,52]. Under sublethal stress, this process is consistent with the “minority MOMP” paradigm, in which limited mitochondrial permeabilization avoids cell death while paradoxically promoting DNA damage and genomic instability [27]. Consistent with this model, inhibition of caspase-8 in our study attenuated EndoG nuclear translocation, suggesting that sublethal caspase-8 activation may indirectly promote EndoG release through limited BID/tBID–BAX/BAK-dependent mitochondrial permeabilization and thereby exacerbate persistent DNA damage. This persistent, sublethal caspase-8 activity bypasses the classical apoptotic cascade, instead fostering chronic genomic instability that establishes a permissive environment for malignant transformation and tumorigenesis. Following DNA damage, early alterations in protein expression dictate cell fate by precisely calibrating the balance between pro-apoptotic and pro-survival programs. NF-κB, a master transcription factor, plays a central role in this process [53]. Interestingly, the biological consequences of genotoxic stress-induced NF-κB activation are highly stimulus-dependent: in specific cell types, UV radiation and doxorubicin treatment shift NF-κB toward transcriptional repression of anti-apoptotic targets, exerting a pro-apoptotic function, whereas etoposide or ionizing radiation promote NF-κB-dependent transcription and reinforce its anti-apoptotic properties [54,55]. Importantly, our findings suggest a potential regulatory link between caspase-8-driven EndoG nuclear enrichment and activation of the non-canonical NF-κB pathway, as reflected by increased expression of p100, p52, and RelB in cells with elevated caspase-8 activity. EndoG has been implicated as an important mediator of persistent radiation-induced DNA damage [28], whereas persistent DNA damage has been shown to activate NIK-dependent RELB/p52 non-canonical NF-κB signaling in another cellular context [56]. Consistent with such a connection, forced nuclear localization of EndoG (NLS-EndoG) in our study was sufficient to enhance p52 and RelB expression. These observations raise the possibility that persistent DNA damage generated following EndoG nuclear translocation serves as an intermediate signal linking caspase-8 activation to non-canonical NF-κB signaling. Such a damage–adaptation coupling may provide a selective survival advantage to genomically damaged cells, allowing them to persist and thereby potentially facilitating long-term genomic instability and malignant transformation. However, because EndoG depletion was not examined in the present study, whether endogenous EndoG is required for caspase-8-mediated p52/RelB activation remains to be established.

This study reveals a non-canonical role for caspase-8 in radiation-induced malignant transformation. Traditionally considered a tumor suppressor, caspase-8—when sublethally activated by ionizing radiation—paradoxically promotes oncogenesis. Mechanistically, sublethal caspase-8 activation promotes EndoG nuclear translocation and persistent DNA damage, accompanied by activation of the pro-survival NF-κB pathway (Figure 7). Notably, although persistent caspase-8-dependent DNA damage was induced following both ^56^Fe ions and X-ray irradiation, under the experimental conditions used in this study, detectable oncogenic transformation was observed only after ^56^Fe ion exposure, which may be related to the more complex and difficult-to-repair DNA damage generated by high-LET ^56^Fe ions [29,30]. These findings may provide further mechanistic insights into the long-term carcinogenic risks associated with deep-space radiation exposure.

## 4. Materials and Methods

### 4.1. Cell Culture

Early-passage immortalized human breast epithelial MCF10A cells were kindly provided by Hatsumi Nagasawa from Colorado State University (Fort Collins, CO, USA). Cells were cultured in growth medium composed of DMEM/F12 supplemented with 5% donor horse serum, 20 ng/mL EGF (R&D systems, Minneapolis, MN, USA), 0.5 μg/mL hydrocortisone (Sigma-Aldrich, St. Louis, MO, USA), 100 ng/mL cholera toxin (Sigma-Aldrich), 10 μg/mL insulin (Invitrogen, Grand Island, NY, USA), and 100 units/mL penicillin and 100 μg/mL streptomycin. For γH2AX foci and comet assay, cells were cultured in an assay medium with only 2% horse serum and without EGF.

### 4.2. Plasmids and Lentivirus Production

A caspase-8 recognition site (IETD) was engineered between a 9-unit polyubiquitin domain and a luciferase–GFP fusion gene; the resulting reporter (Casp8-GFP-Luc) was constructed into the lentiviral vector pLEX (Open Biosystem, Huntsville, AL, USA). The human dominant-negative caspase-8 (Casp8DN, C360A) was obtained from Addgene and cloned into pLEX with a hemagglutinin (HA) tag at the 3′ end. The shRNA sequence targeting *caspase-8* (shCASP8, CACCAGGCAGGGCTCAAATTT) was constructed in pLKO.1-EGFP-PURO. Nuclear-localized EndoG (NLS-EndoG) was generated as previously described [36] and inserted into a lentiviral vector.

Lentivirus was produced by transfecting 293T cells with the lentiviral vectors and second-generation packaging plasmids (psPAX2, pMD2.G) following published protocols (http://tronolab.epfl.ch/lentivectors (accessed on 16 August 2026)). Stable cell lines were established by lentiviral transduction and antibiotic selection.

### 4.3. Animal Models

Caspase-8 heterozygous (*Casp8*^+/−^) and age-matched wild-type (WT) C57BL/6JCya mice, including both males and females, aged 6–8 weeks and weighing 20–25 g, were obtained from Cyagen Biosciences (Guangzhou, China). Mice were assigned to four groups: non-irradiated WT, irradiated WT, non-irradiated *Casp8*^+/−^, and irradiated *Casp8*^+/−^ mice (*n* = 3 per group). Mice in the irradiated groups received a single 3 Gy whole-body X-ray irradiation using a PXi X-RAD 225 irradiator (Cancer Research Institute and Cancer Hospital, Guangzhou Medical University, Precision X-Ray, Inc., North Branford, CT, USA). The 3 Gy dose was selected based on established whole-body X-ray irradiation models showing persistent chromosomal abnormalities and genomic instability in mouse bone marrow [28,57].

### 4.4. Radiation Exposure

MCF10A cells (sealed in T-25 flasks) were exposed to high-energy ^56^Fe ions (600 MeV/n, dose rate: 0.5 Gy/min) at the National Aeronautics and Space Administration (NASA)-sponsored Space Radiation Laboratory at Brookhaven National Laboratory (BNL; Brookhaven, Long Island, NY, USA), or X-rays in a PXi X-RAD 225 irradiator (225 kV, 13.3 mA, dose rate: 2 Gy/min, Cancer Research Institute and Cancer Hospital, Guangzhou Medical University). The irradiation doses were selected based on previously established conditions in MCF10A cells. Sub-Gy ^56^Fe-ion irradiation has been shown to induce cytogenetic damage, and 0.5 Gy ^56^Fe-ion exposure can induce persistent DNA damage and genomic instability in surviving cells [28,58]. In comparison, approximately 5–6 Gy of low-LET photon irradiation has been used to investigate DNA damage responses and genomic stability in the same cell system [59,60,61].

### 4.5. Monitoring Caspase-8 Reporter Activation

Post-irradiation, caspase-8 reporter cells were plated at 1 × 10^4^ cells/well in 6-well plates. Reporter activity was measured using a Synergy H1 Hybrid Multi-Mode Microplate Reader (BioTek, Winooski, VT, USA) with 150 μg/mL luciferin, and luciferase activity was quantified with the manufacturer’s software. In separate experiments, irradiated MCF10A cells (0.5 Gy) carrying the caspase-8 reporter were sorted by FACS (BD FACSVantage SE, Milpitas, CA, USA) into GFP-high and GFP-low populations.

### 4.6. γH2AX Foci Assay

To detect the radiation-induced DNA double-strand breaks, γH2AX foci in the irradiated cells were examined through immunofluorescence using an established protocol [62]. Irradiated or non-irradiated MCF10A cells were seeded in culture dishes, cultured with growth medium overnight, then changed to assay medium for another 72 h. Cells were fixed, permeabilized, blocked and incubated with an anti-γH2AX primary antibody (Upstate Biotechnology, Lake Placid, NY, USA), followed by Alexa Fluor 488- or 555-conjugated secondary antibodies (Invitrogen, Carlsbad, CA, USA). After mounting with DAPI-containing mounting medium, images were captured using a TCS SP5 inverted confocal microscope (Leica Microsystems GmbH, Wetzlar, Germany) with a 63× oil immersion objective and analyzed using ImageJ software (version 1.49p).

### 4.7. Alkaline Comet Assay

The alkaline comet assay [63,64,65] was conducted to detect DNA strand breaks. Following the manufacturers’ protocols (Trevigen, Gaithersburg, MD, USA; Beyotime, Shanghai, China), cells were mixed with low-melting agarose, spread on slides, and solidified on ice. After lysis in cold buffer and DNA unwinding at room temperature, alkaline electrophoresis was performed. The cells were then neutralized, stained with fluorescent dye and examined by fluorescence microscopy (Leica Microsystems GmbH, Wetzlar, Germany) at 20× magnification. Comet images were captured, and the percentage of tail DNA was quantified to reflect DNA damage. Comet images were analyzed using CASP Lab (version 1.2.3 beta 1).

### 4.8. Bone Marrow Isolation and Flow Cytometric Analysis of γH2AX

Irradiated mice were euthanized 6 days after irradiation, while non-irradiated controls were euthanized at the corresponding time point. Bone marrow cells were flushed from femurs and tibias with PBS, lysed with RBC lysis buffer (Beyotime, Cat#C3702) on ice, and washed with PBS containing 2% FBS. A total of 5 × 10^6^ cells were fixed (4% PFA), permeabilized (0.1% Triton X-100), and blocked. Cells were incubated with anti-γH2AX primary antibody overnight at 4 °C, then with fluorescent secondary antibody for 1 h at room temperature (RT). After washing, cells were analyzed by flow cytometry (CytoFLEX, Beckman Coulter, Brea, CA, USA), and data were analyzed using the FlowJo software (version 10.8.1).

### 4.9. Chromosome Aberration Analysis

Fluorescence in situ hybridization (FISH) was performed on bone marrow cells from non-irradiated and irradiated caspase-8 wild-type (WT) or heterozygous (*Casp8*^+/−^) mice. Whole-chromosome painting probes for mouse chromosome 1 (XMP 1 Green) and chromosome 2 (XMP 2 Orange) (MetaSystems, Altlussheim, Germany) were used to analyze chromosomal translocations.

### 4.10. Western Blot Analysis

Cells were lysed in RIPA buffer supplemented with protease inhibitors. Equal amounts of protein were separated by SDS-PAGE. See Supplementary Materials Table S1 for information on the antibodies.

### 4.11. Soft-Agar Assay

To measure the ability of growth in 3D, a soft agar assay was performed according to an established protocol. Irradiated MCF10A cells were seeded at a density of 5000 per well in 6-well plates in triplicate. After growth in the plates for three to four weeks, the colonies were fixed and stained with 0.005% crystal violet.

### 4.12. In Vivo Tumor Growth

To confirm the tumorigenicity of irradiated MCF10A cells, 2 × 10^6^ iron-ion-irradiated cells were injected subcutaneously into the hind limb of 6–8-week-old female nude mice, which were purchased from The Jackson Laboratory (Bar Harbor, ME, USA). The mice were monitored for tumor growth every two weeks for 12 weeks. Tumor volume was calculated as (length × width^2^)/2. Tumors were harvested at the end of the study for Hematoxylin and Eosin (H&E) staining. Animals were euthanized via carbon dioxide (CO_2_) inhalation using a gradual-fill method (displacement rate of 30–70% chamber volume/min), consistent with AVMA guidelines. CO_2_ flow was maintained for at least 1 min after respiratory arrest, followed by cervical dislocation as a secondary physical method to ensure death, in full compliance with IACUC protocols.

### 4.13. Immunofluorescence Analysis

Cells grown in culture dishes were fixed with 4% paraformaldehyde, followed by permeabilization with 0.25% Triton X-100. To quench endogenous GFP fluorescence, the cells were then incubated with Autofluorescence Quencher (Applygen, Beijing, China, Cat# C1212) according to the manufacturer’s instructions. After washing, cells were blocked with blocking buffer (5% normal goat serum) for 30 min at room temperature. Cells were incubated with primary antibodies overnight at 4 °C, followed by incubation with appropriate Alexa Fluor 488- or 555-conjugated secondary antibodies for 1 h at RT and mounted with mounting medium containing DAPI. See Supplementary Materials Table S1 for information on the antibodies. Fluorescent images were captured using a confocal microscope.

### 4.14. RNA-Sequencing and Data Analysis

Total RNA was extracted using TRIzol and sequenced by BGI. mRNA was enriched using Oligo(dT) beads, fragmented, and reverse-transcribed to cDNA. The library, constructed through end repair, A-tailing, adapter ligation, and PCR, was validated using a Qubit fluorometer (Thermo Fisher Scientific, Eugene, OR, USA) and an Agilent 2100 Bioanalyzer (Agilent Technologies, Inc., Santa Clara, CA, USA) before 150 bp paired-end sequencing on the DNBSEQ platform. Clean reads were mapped to the reference genome using HISAT2. Differentially expressed genes (DEGs) were identified using DESeq2 with thresholds of |log2FC| > 1 and adjusted *p* < 0.05. KEGG pathway enrichment analysis was subsequently performed to identify significantly enriched pathways among the DEGs.

### 4.15. Statistical Analysis

Data were presented as mean ± SEM. Statistical significance between two groups was determined using the unpaired Student’s *t*-test. A *p* value less than 0.05 was considered statistically significant. Statistical analyses were performed using GraphPad Prism (version 9.0).

## Data Availability

The original contributions presented in this study are included in the article/Supplementary Material. Further inquiries can be directed to the corresponding author.

## Supplementary Materials

The following supporting information can be downloaded at https://www.mdpi.com/article/10.3390/ijms27167398/s1.

## Abbreviations

The following abbreviations are used in this manuscript:

Casp8DN	Dominant-negative caspase-8
TRAIL	TNF-related apoptosis-inducing ligand
IETD	Isoleucine-glutamic acid-threonine-aspartic acid
DSBs	DNA double-strand breaks
DEGs	Differentially expressed genes
