# Supplementary material for: Sublethal Caspase-8 Activation Drives Radiation-Induced Genetic Instability and Oncogenic Transformation Through Endonuclease G and Non-Canonical NF-κB Signaling
Source: Int J Mol Sci. 2026 Aug 19;27(16):7398. doi: 10.3390/ijms27167398 (PMC13513489; doi:10.3390/ijms27167398)
Supplement: Supplementary file 1 [file ijms-27-07398-s001.zip › ijms-4476991-supplementary.pdf]

Sublethal Caspase-8 Activation Drives Radiation-induced Genetic Instability and Oncogenic Transformation through Endonuclease G and Non-Canonical NF-κB Signaling

Chenchen Zhu[1*], Xiaoxiao Li[2], Chuan-Yuan Li[2], Renwei Liu[1], Yifei Wang[1]

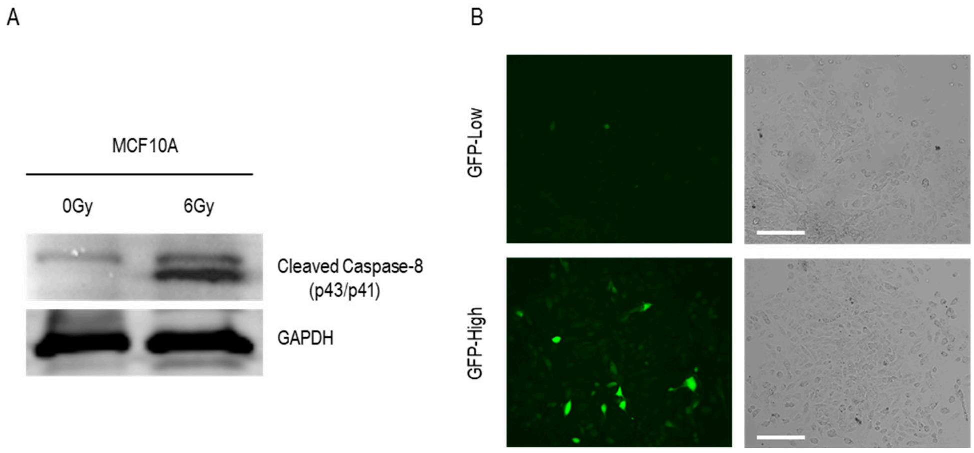

Figure S1 X-ray irradiation induces both caspase-8 cleavage and EGFP reporter activity in MCF10A cells.

(A) Western blot analysis of cleaved caspase-8 in MCF10A cells at 10 days after 6 Gy X-ray irradiation. GAPDH was used as a loading control.

(B) Cell populations with low (top panel) and high (lower panel) EGFP expression from X-ray irradiated (6 Gy) MCF10A cells were separated by flow cytometry at 7 days after irradiation. Scale bars represent 100 μm.

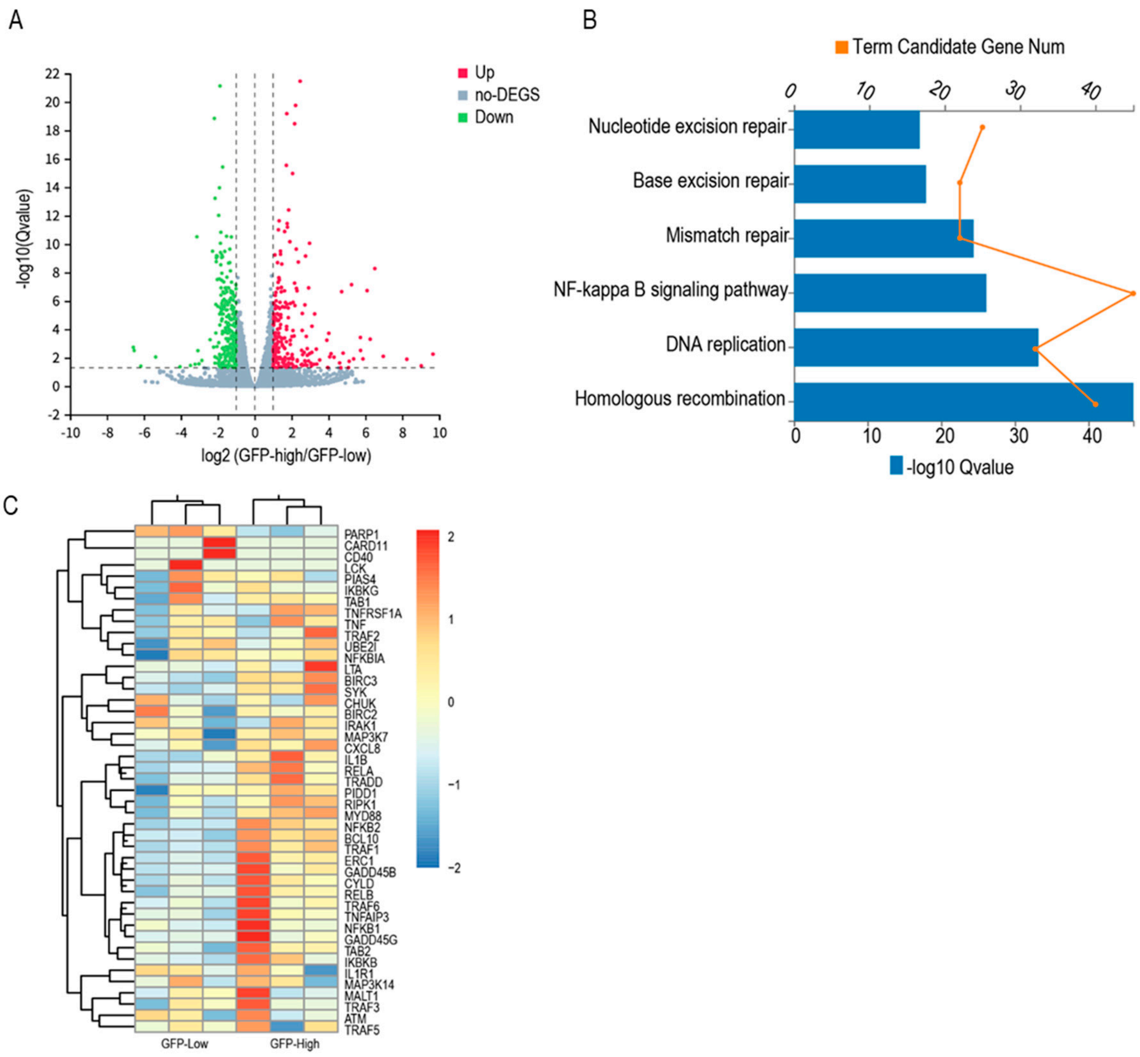

Figure S2 Volcano plot of differentially expressed genes and KEGG pathway enrichment in GFP-low vs. GFP-high MCF10A cells.

(A) Volcano plot of differentially expressed genes (DEGs) between GFP-low and GFP-high MCF10A cells.

(B) KEGG pathway enrichment analysis of DEGs, showing pathways including mismatch repair (MMR), NF-κB signaling pathway DNA replication, homologous recombination (HR).

(C) The heatmap displays a complete list of 45 DEGs belonging to the KEGG NF-κB signaling pathway, differentially expressed between high and low GFP-expressing subpopulations isolated from irradiated MCF10A cells.

Table S1. Antibodies used in this study

Antibodies	Source	Identifier	Dilution
mouse anti-phospho-Histone H2A.X (Ser139)	Sigma-Aldrich	Cat#05-636	1:1000 for WB
mouse anti-ENDOG (Endonuclease G)	ORIGENE	Cat#TA504189; RRID: AB_11124573	1:1000 for WB, 1:100 for IF
mouse anti-GAPDH	Proteintech	Cat#60004-1-Ig; RRID: AB_2107436	1:20000 for WB
mouse anti-β-Actin	Proteintech	Cat#66009-1-Ig; RRID: AB_2687938	1:20000 for WB
rabbit anti-Caspase-8 (D35G2)	Cell Signaling Technology	Cat# 4790T; RRID: AB_10545768	1:1000 for WB
rabbit anti-Cleaved Caspase-8 (Asp374)	Cell Signaling Technology	Cat# 98134S; RRID:AB_3107200	1:1000 for WB
rabbit anti-HA-Tag	Cell Signaling Technology	Cat# 3724S; RRID; AB_1549585	1:1000 for WB
rabbit anti-NF-kappaB2 p100/p52	Cell Signaling Technology	Cat# 4882T; RRID; AB_10695537	1:1000 for WB
rabbit anti-RelB	Cell Signaling Technology	Cat# 4922T; RRID; AB_2179173	1:1000 for WB
IRDye® 800CW Goat Anti-Mouse IgG	LICORbio	Cat#926-32210	1:10000 for WB
IRDye 680RD Goat anti-Rabbit IgG	LICORbio	Cat#926-68071	1:10000 for WB
Goat anti-Rabbit IgG (H+L) Cross-Adsorbed Secondary Antibody, Alexa Fluor™ 405 - A-31556	Thermo Fisher	Cat#A21428	1:500 for IF
Goat anti-Mouse IgG (H+L) Cross-Adsorbed Secondary Antibody, Alexa Fluor™ 555	Thermo Fisher	Cat#A21422	1:500 for IF
Goat anti-Mouse IgG (H+L) Cross-Adsorbed Secondary Antibody, Alexa Fluor™ 488	Thermo Fisher	Cat# A11001	1:2000 for IF

WB: Western blot, IF: Immunofluorescence
